# Molecular Detection and Genomic Characterization of Porcine Enterovirus G in Guangxi, China: Genotype Diversity, PLCP Insertions, and Recombination

**DOI:** 10.3390/v18070707

**Published:** 2026-06-26

**Authors:** Kaiyi Jiang, Bin Li, Xianhua Wu, Wen Zhao, Yibin Qin, Shuo Zhao, Zhongwei Chen, Wenfeng Wang, Qunpeng Duan, Yingning Zhou, Chenyu Quan, Xinting Xu, Tingting Chen, Yilan Xu, Huimei Su, Xunye Yang, Yang Qin, Ying Peng, Ying He, Bingxia Lu

**Affiliations:** 1 College of Animal Science and Technology, Guangxi University, Nanning 530004, China; 2 Key Laboratory of Veterinary Biotechnology of Guangxi, Key Laboratory of China (Guangxi)-ASEAN Cross-Border Animal Disease Prevention and Control (Ministry of Agriculture and Rural Affairs of China), Guangxi Veterinary Research Institute, Nanning 530001, China; 3 Shengtang Animal Husbandry Co., Ltd., Guangxi Nongken Yongxin Animal Husbandry Group, Liuzhou 545211, China

**Keywords:** Enterovirus G, PLCP gene insertion, recombination, genetic diversity, phylogenetic analysis

## Abstract

Enterovirus G (EV-G) is an important enteric pathogen widely circulating in swine populations and is characterized by considerable genetic diversity and recombination potential. In recent years, recombinant EV-G strains carrying exogenous papain-like cysteine protease (PLCP) gene insertions have been increasingly reported; however, their genotype distribution and molecular characteristics in major pig-producing regions remain poorly understood. In this study, 356 clinical samples collected from Guangxi, southern China, between 2020 and 2025 were screened for EV-G, and 13 representative strains were subjected to whole-genome sequencing and sequence analysis. The overall EV-G positivity rate in Guangxi was 20.51% (73/356). Phylogenetic analysis showed that the 13 Guangxi EV-G strains were mainly classified into three genotypes, G1, G2, and G8, with G1 being the predominant genotype. Notably, PLCP gene insertions of 573–642 nt were identified at the 2C/3A junction in seven strains belonging to three distinct genotypes, G1, G2, and G8, demonstrating the cross-genotype distribution of PLCP insertions within a single geographic region. Phylogenetic analysis of the PLCP sequences demonstrated that all Guangxi-derived PLCP sequences clustered within the EV-G-PLCP clade and were clearly separated from the torovirus PLCP clade. Recombination analysis retained three potential recombination events with clearer combined support from RDP4 and SimPlot analyses, involving Guangxi strains GX3008, GX3022, and GX4292. Selection pressure analysis showed that the VP1 gene was overall under negative selection. Collectively, these findings demonstrate the co-circulation of multiple EV-G genotypes, the cross-genotype distribution of PLCP insertions, and the presence of potential recombination events in Guangxi. This study provides new evidence for understanding the genetic diversity, genomic plasticity, and regional molecular characteristics of EV-G, and also provides an important basis for future PLCP-related functional studies and continued EV-G surveillance.

## 1. Introduction

Enterovirus G (EV-G), a member of the genus Enterovirus in the family Picornaviridae, is a non-enveloped, positive-sense single-stranded RNA virus with a genome of approximately 7.4 kb. Its genome encodes a polyprotein precursor that is proteolytically cleaved into four structural proteins (VP1–VP4) and seven non-structural proteins (2A^pro–3D^pol) [1,2,3]. Among these, the capsid protein VP1 constitutes the major antigenic region and serves as an important molecular marker for EV-G genotyping [4,5,6], whereas the non-structural proteins 2C and 3A play critical roles in viral replication and virus–host interactions. To date, 20 EV-G genotypes (G1–G20) have been identified, reflecting the substantial genetic diversity of this virus [5,7,8,9].

Clinically, EV-G infection is mainly associated with enteric disease in swine and commonly presents as post-weaning diarrhea [1,10,11], often accompanied by pathological changes such as villous atrophy, crypt hyperplasia, and intestinal barrier damage [12]. Over the past decades, EV-G has been widely reported in swine populations across Europe, East Asia, and Southeast Asia [5,8,13]. Molecular epidemiological studies have shown that multiple EV-G genotypes can co-circulate within the same region, and that recombination and exogenous sequence insertion are important contributors to EV-G genomic diversity [5,6,8].

In recent years, insertion events within the non-structural protein-coding region of EV-G have attracted increasing attention. In particular, insertion of a papain-like cysteine protease (PLCP) gene into the relatively conserved 2C/3A junction region has been reported in multiple EV-G lineages and is considered one of the important features associated with EV-G genomic plasticity [3,5,11]. Previous studies have shown that PLCP is derived from toroviruses and possesses deubiquitinating and deISGylating activities, which may influence the interaction between the virus and the host innate immune response [14,15]. Although an increasing number of EV-G strains carrying PLCP insertions have been reported, systematic studies addressing their genotype distribution, molecular characteristics, and regional genetic features within different pig-producing regions, particularly based on multiple genotypes circulating within the same region, remain limited.

At present, knowledge regarding EV-G genetic diversity and molecular characteristics remains uneven across different geographic regions worldwide. PLCP insertions were first identified in EV-G strains from the United States and Europe and were subsequently reported in strains belonging to genotypes G1, G2, G8, G10, and G17 in Korea, Japan, China, and other regions [5,7,9,11,16,17]. However, existing studies have largely focused on single or only a few genotypes, and systematic comparisons of multi-genotype EV-G strains and their PLCP insertion events within the same geographic region are still lacking. This has, to some extent, limited a deeper understanding of the cross-genotype molecular characteristics of EV-G [5,7,8,11].

Guangxi, located in southern China, is one of the country’s major pig-producing regions and is geographically adjacent to Southeast Asia, with relatively frequent movement of animals and animal products. This provides an important epidemiological background for investigating the molecular epidemiology, recombination signals, and regional molecular characteristics of EV-G. Previous studies have reported widespread EV-G circulation in Guangxi and have identified recombinant strains carrying PLCP gene insertions [4,18,19]. However, it remains unclear whether PLCP insertion events are widely distributed across different genotype backgrounds in this region, and comprehensive analyses of their molecular characteristics, phylogenetic relationships, and associated genomic features are still lacking.

Therefore, in the present study, clinical samples collected from multiple regions of Guangxi, southern China, between 2020 and 2025 were screened for EV-G, and representative circulating strains were subjected to whole-genome sequencing and sequence analysis. The aims of this study were to determine the genotype distribution and genomic diversity of EV-G in Guangxi, to clarify the genotype range and molecular characteristics associated with PLCP insertions, and to investigate EV-G genetic characteristics under different genotype backgrounds through phylogenetic, recombination, and selection pressure analyses. These findings are expected to provide a basis for further understanding the genetic diversification, genomic plasticity, and regional molecular characteristics of EV-G.

## 2. Materials and Methods

### 2.1. Materials

#### 2.1.1. Clinical Samples

From 2020 to 2025, a total of 356 clinical samples were collected from pigs with suspected enteric viral infection or diarrhea-related clinical signs in Guangxi, southern China. These samples were submitted to our laboratory for routine diagnostic testing and molecular surveillance. The samples were obtained from 11 regions of Guangxi, including Beihai, Chongzuo, Fangchenggang, Guigang, Guilin, Hechi, Laibin, Liuzhou, Nanning, Qinzhou, and Yulin. The sample types included intestinal tissues, intestinal contents, anal swabs, and fecal samples. Because these samples were derived from routine clinical submissions rather than from a standardized population-based sampling program, detailed metadata such as pig age, farm-level sampling structure, and herd size were not consistently available for all samples.

#### 2.1.2. Reagents

The magnetic bead-based nucleic acid extraction kit was purchased from Shenzhen Haiteng Biotechnology Co., Ltd. (Shenzhen, China); M-MLV reverse transcriptase was purchased from Thermo Fisher Scientific (Waltham, MA, USA); 2× Bench Top Taq Premix (AT1201) was purchased from Hangzhou Bovow Medical Technology Co., Ltd. (Hangzhou, China); PrimeSTAR Max DNA Polymerase (R045A) was purchased from Takara Bio Inc. (Shiga, Japan); and the D2000 Plus DNA marker was purchased from Beijing Kangrun Chengye Biotechnology Co., Ltd. (Beijing, China).

#### 2.1.3. Instruments

The instruments used in this study included a gradient polymerase chain reaction (PCR) thermal cycler (T100, Bio-Rad, Hercules, CA, USA), an electrophoresis apparatus (Beijing Liuyi Biotechnology Co., Ltd., Beijing, China), and a gel imaging system (Universal Hood II, Bio-Rad, Hercules, CA, USA).

#### 2.1.4. Primers

According to previously reported methods [20,21], one pair of PCR primers was designed and synthesized for the clinical detection of porcine EV-G (Table 1). This primer pair targets a conserved fragment within the EV-G 5′ untranslated region (5′ UTR; also referred to as the 5′ non-coding region or 5′ non-translated region) and amplifies an EV-G-specific fragment of approximately 314 bp. The primers were synthesized by Sangon Biotech Co., Ltd. (Shanghai, China) and diluted with double-distilled water (ddH_2_O) to a working concentration of 25 μM before use.

### 2.2. Methods

#### 2.2.1. Viral Nucleic Acid Extraction

Clinical samples collected from diseased pigs, including minced and homogenized intestinal tissues, intestinal contents, anal swabs, and fecal samples, were thoroughly mixed with sterile normal saline, subjected to three freeze–thaw cycles, and then centrifuged at 10,000× *g* for 3 min. A total of 200 μL of the supernatant was collected, and viral nucleic acids were extracted according to the manufacturer’s instructions of the magnetic bead-based nucleic acid extraction kit. The extracted viral RNA was immediately used for subsequent reverse transcription and PCR amplification.

#### 2.2.2. Reverse Transcription (RT)

The reverse transcription reaction was performed in a total volume of 12.5 μL, containing 2.5 μL of 5× M-MLV reverse transcriptase buffer, 1.0 μL of dNTP mixture, 0.25 μL of M-MLV reverse transcriptase, 0.25 μL of Ribolock RNase inhibitor, 0.5 μL of the reverse primer (working concentration: 25 μM), and 8.0 μL of viral RNA template. The reverse transcription reaction was carried out at 42 °C for 1 h.

#### 2.2.3. PCR Amplification

1. PCR Assay for EV-G Detection

The PCR amplification reaction used for EV-G detection was performed in a total volume of 25 μL, containing 8.5 μL of double-distilled water (ddH_2_O), 12.5 μL of 2× Bench Top Taq Premix, 0.5 μL each of the forward and reverse primers (EV-G-F and EV-G-R), and 3 μL of complementary DNA (cDNA) template. The PCR cycling conditions were as follows: initial denaturation at 94 °C for 5 min; 35 cycles of denaturation at 94 °C for 30 s, annealing at 53 °C for 40 s, and extension at 72 °C for 30 s; followed by a final extension at 72 °C for 10 min [13].

2. Agarose Gel Electrophoresis

A total of 5 μL of the PCR products was subjected to electrophoresis on a 1.0% (*w*/*v*) agarose gel. After electrophoresis, the amplified products were visualized and photographed using a gel imaging system.

#### 2.2.4. Whole-Genome Sequencing of EV-G

Thirteen EV-G-positive samples were included in the whole-genome analysis based on quality and completeness criteria, including reverse transcription-polymerase chain reaction (RT-PCR) positivity, sample availability, sampling year, geographic source, sample type, nucleic-acid quality, sequencing coverage, and successful complete-genome assembly. These samples were used for subsequent whole-genome characterization, phylogenetic analysis, PLCP insertion analysis, and recombination analysis. Viral nucleic acids from these 13 EV-G-positive samples were processed by Shanghai Tanpu Biotechnology Co., Ltd. (Shanghai, China) for library construction and high-throughput sequencing. Briefly, viral RNA was reverse-transcribed using random hexamers, followed by second-strand synthesis, and libraries were prepared using the Nextera XT DNA Library Preparation Kit (Illumina, Inc., San Diego, CA, USA). Sequencing was performed on the Illumina NovaSeq 6000 platform (Illumina, Inc., San Diego, CA, USA) using a paired-end 150-nt sequencing strategy. Raw sequencing reads were filtered using fastp v0.20.0 to remove adapter sequences and low-quality reads, including reads with quality scores below Q20. Host-, ribosomal RNA (rRNA)-, and bacterial-derived reads were removed using BBmap v38.51 by comparison with ribosomal RNA sequences, bacterial genome sequences, and the host genome. The remaining clean reads were assembled de novo using SPAdes v3.13.0. The final contigs were filtered using a minimum length threshold of 100 bp and subjected to BLAST+ v2.10.0 homology searches against the National Center for Biotechnology Information (NCBI) nucleotide (NT) database to confirm sequence identity, genome orientation, and genome coverage. Preliminary coding sequence prediction was performed using Prokka v1.14.5, followed by manual correction and annotation according to EV-G reference genome organization, including the 5′ non-coding region, polyprotein-coding region, and 3′ non-coding region. The final complete genome sequences were submitted to the GenBank database, where accession numbers were assigned.

#### 2.2.5. Phylogenetic Analysis

1. Phylogenetic Analysis Based on the VP1 Gene

A total of 79 complete VP1 coding-region nucleotide sequences, including 13 EV-G strains identified in Guangxi and 66 reference strains representing genotypes EV-G1 to EV-G20, were used for phylogenetic analysis. Multiple sequence alignment was performed using MAFFT v7.526. Based on the aligned nucleotide sequences, a phylogenetic tree was constructed using the Maximum-Likelihood (ML) method implemented in MEGA 12 under the GTR+G+I model. Branch support was evaluated by 1000 bootstrap replicates, and only bootstrap values greater than 70% were displayed. Reference sequences representing EV-G1 to EV-G20 were retrieved from the GenBank database based on previously reported genotype assignments and sequence availability [1,22].

2. Phylogenetic Analysis Based on the Complete Genome

Complete genome nucleotide sequences of 13 Guangxi EV-G strains and 34 EV-G reference strains were used for phylogenetic analysis. Multiple sequence alignment was performed using MAFFT v7.526. Based on the aligned complete genome nucleotide sequences, a phylogenetic tree was constructed using the ML method implemented in MEGA 12 under the GTR+G+I model, and branch support was assessed by 1000 bootstrap replicates. By comparing the topological differences between the VP1-based phylogenetic tree and the complete genome-based phylogenetic tree, the clustering characteristics of different strains in the two trees were analyzed to provide evidence for subsequent recombination analysis. The 34 EV-G reference complete-genome sequences were retrieved from the GenBank database based on genome completeness, genotype information, and relevance to the strains analyzed in this study.

#### 2.2.6. Sequence Homology Analysis

The complete genome sequences, polyprotein-coding sequences, and VP1 gene sequences of the 13 EV-G strains identified in Guangxi were subjected to sequence homology analysis. Multiple sequence alignment was performed using MAFFT v7.526. For complete-genome alignments, insertion regions and alignment gaps were retained to evaluate genome-structure differences, particularly PLCP insertion-associated length variation. For polyprotein-coding sequences and VP1 coding sequences, the corresponding coding regions were extracted according to genome annotation, and the reading frame was maintained during alignment and translation. The alignments were manually inspected before sequence identity calculation to avoid the influence of obvious terminal gaps or alignment errors. Nucleotide and amino acid sequence identities were then calculated using BioAider v1.727 based on the inspected alignments [21].

#### 2.2.7. Analysis of PLCP Insertion Sequences

Multiple sequence alignment was performed using MAFFT v7.526 to compare the complete genome sequences of the 13 Guangxi EV-G strains with EV-G reference sequences with and without previously reported PLCP insertions. The 2C/3A junction region was located according to the annotated polyprotein-coding region and by comparison with non-PLCP EV-G reference genomes. PLCP insertion sequences were identified as additional nucleotide fragments present at the 2C/3A junction in Guangxi EV-G strains relative to non-PLCP EV-G reference genomes and showing sequence similarity to previously reported EV-G-PLCP insertion sequences. The insertion boundaries were determined based on the alignment positions immediately flanking the inserted fragment at the 2C/3A junction, and insertion lengths were calculated according to the number of nucleotides within the inserted region. Alignment gaps introduced to accommodate PLCP insertion fragments were retained during complete-genome comparison and insertion-boundary determination. The putative PLCP insertion sequences were extracted, translated into deduced amino acid sequences, and compared with previously reported EV-G-PLCP sequences and corresponding PLCP sequences from porcine torovirus (PToV) and bovine torovirus (BToV) reference strains to evaluate their PLCP-like features. Nucleotide and deduced amino acid sequence identities were calculated among Guangxi EV-G-PLCP strains, between Guangxi EV-G-PLCP strains and previously reported EV-G-PLCP reference strains, and between Guangxi EV-G-PLCP strains and PToV/BToV PLCP reference sequences. For PLCP amino acid alignment, gaps introduced by length variation among PLCP insertions were retained for visual comparison. The PLCP amino acid alignment was generated in MEGA 12 based on the MAFFT alignment output. For visualization, the complete amino acid sequence of strain GX3008 was displayed as the reference sequence, whereas dots in the remaining sequences indicate amino acid residues identical to those of GX3008. The sequences were grouped as Guangxi EV-G-PLCP strains, EV-G-PLCP reference strains, PToV strains, and BToV strains.

Based on the deduced amino acid sequences of PLCP, multiple sequence alignment was carried out using MAFFT v7.526, and a phylogenetic tree was constructed using the ML method implemented in MEGA 12 under the JTT+G model. Branch support was evaluated using 1000 bootstrap replicates, and only bootstrap values greater than 70% were displayed. Previously reported EV-G-PLCP, porcine torovirus, and bovine torovirus PLCP reference sequences were retrieved from the GenBank database for comparative and phylogenetic analyses.

#### 2.2.8. Recombination Analysis

Complete genome sequences of the 13 Guangxi EV-G strains together with those of 34 EV-G reference strains retrieved from the GenBank database were used for recombination analysis. Potential recombination events were initially detected using seven methods implemented in RDP4 v4.101, namely RDP, Chimaera, BootScan, 3Seq, GENECONV, MaxChi, and SiScan [23]. Events supported by multiple methods with a significance level of *p* < 0.05 were considered potential recombination events. The 5′ breakpoint, 3′ breakpoint, and putative recombinant region of each event were determined with reference to the RDP4 output.

Potential recombination events were further evaluated using SimPlot v3.5.1, with a sliding window size of 200 bp and a step size of 20 bp, to analyze nucleotide similarity changes across the genome and to assess whether the inferred recombinant regions were consistent with parental-similarity shifts in the corresponding SimPlot plots [24]. Events showing consistent support from RDP4 and SimPlot analyses were retained for final recombination interpretation.

#### 2.2.9. Selection Pressure Analysis of the VP1 Gene

A total of 79 complete VP1 coding-region sequences were included in the selection pressure analysis, including 13 Guangxi EV-G strains identified in this study and 66 reference strains representing genotypes EV-G1 to EV-G20. VP1 was selected because it is the major coding region commonly used for EV-G genotyping and genetic diversity assessment. Multiple sequence alignment was performed at the codon level, and the reading frame was maintained before downstream selection pressure analysis. Site-specific selection pressure was analyzed using the SLAC, FEL, FUBAR, and MEME methods implemented in HYPHY v2.5.70 to identify potential positively selected sites [25,26]. In addition, DnaSP v6.12.03 was used to calculate the nonsynonymous substitution rate (dN), synonymous substitution rate (dS), and their ratio ω (dN/dS). An ω value greater than 1 indicates positive selection, an ω value less than 1 indicates negative (purifying) selection, and an ω value equal to 1 indicates neutral evolution [27].

## 3. Results

### 3.1. Agarose Gel Electrophoresis of EV-G RT-PCR Products

**Figure 1:**
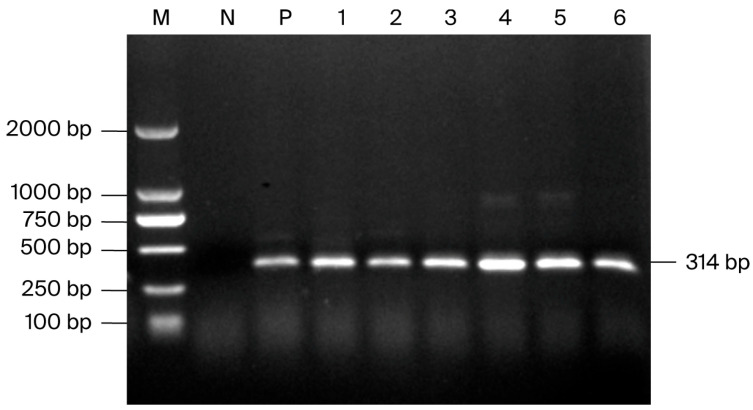
Agarose gel electrophoresis of RT-PCR products for EV-G detection in clinical samples from Guangxi. M, DNA marker DL2000; N, negative control (ddH_2_O); P, EV-G-positive control; lanes 1–6, clinical EV-G-positive samples.

Following RT-PCR detection of clinical samples collected from 11 regions of Guangxi between 2020 and 2025, representative EV-G-positive samples all yielded the expected target band of approximately 314 bp (Figure 1), indicating successful amplification of the target fragment.

### 3.2. RT-PCR Detection of EV-G in Clinical Samples from Guangxi

From 2020 to 2025, a total of 356 clinical samples from 11 regions of Guangxi were tested for EV-G by RT-PCR. Among these samples, 73 were positive for EV-G, giving an overall detection rate of 20.51% (73/356). EV-G-positive samples were detected in nine of the 11 surveyed regions (Table 2).

### 3.3. Genomic Characteristics of the 13 Guangxi EV-G Strains

High-throughput sequencing was performed for 13 EV-G-positive samples from Guangxi, and complete genome sequences were successfully obtained for all strains. The genome lengths ranged from 7354 to 8033 nt. Seven strains, GX3008, GX4181, GX4262A, GX4262C, GX4268, GX4281, and GX4350, harbored PLCP insertions of 573–642 nt. These PLCP-positive strains belonged to genotypes G1, G2, and G8. The sampling information, genotype assignments, genome lengths, PLCP insertion characteristics, and GenBank accession numbers of the 13 strains are summarized below (Table 3).

### 3.4. Sequence Homology Analysis of 13 Guangxi EV-G Strains

Sequence homology analysis of the complete genome sequences of the 13 Guangxi EV-G strains showed that the nucleotide identities ranged from 69.50% to 99.89%. Among them, GX4251A and GX4350 exhibited the lowest identity (69.50%), whereas GX3047A and GX3047B showed the highest identity (99.89%).

Homology analysis of the polyprotein-coding sequences of the 13 Guangxi EV-G strains showed that the nucleotide identities ranged from 70.89% to 99.89%. GX4251A and GX4350 displayed the lowest nucleotide identity (70.89%), whereas GX3047A and GX3047B showed the highest nucleotide identity (99.89%). The deduced amino acid identities of the polyprotein ranged from 75.82% to 99.91%, with the lowest identity observed between GX4251A and GX4350 (75.82%) and the highest identity observed between GX3047A and GX3047B (99.91%).

Homology analysis of the VP1 gene sequences of the 13 Guangxi EV-G strains showed that the nucleotide identities ranged from 55.75% to 100%. The lowest nucleotide identity was observed between GX3004 and GX4268 (55.75%), whereas the highest was observed between GX3047A and GX3047B (100%). The deduced amino acid identities of VP1 ranged from 52.68% to 100%, with the lowest identity observed between GX3008 and GX4292 (52.68%) and the highest between GX3047A and GX3047B (100%). Further sequence alignment revealed a total of 59 variable amino acid sites in the VP1 protein among the 13 Guangxi EV-G strains.

### 3.5. Phylogenetic Relationships of Guangxi EV-G Strains

#### 3.5.1. Phylogenetic Analysis Based on the VP1 Gene

**Figure 2:**
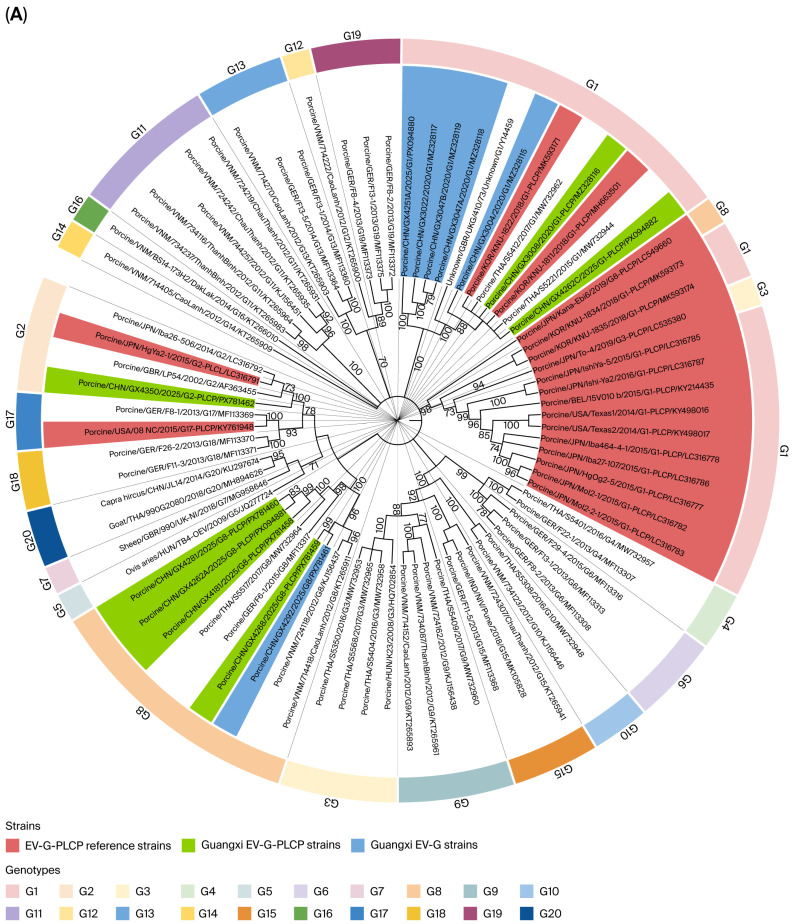

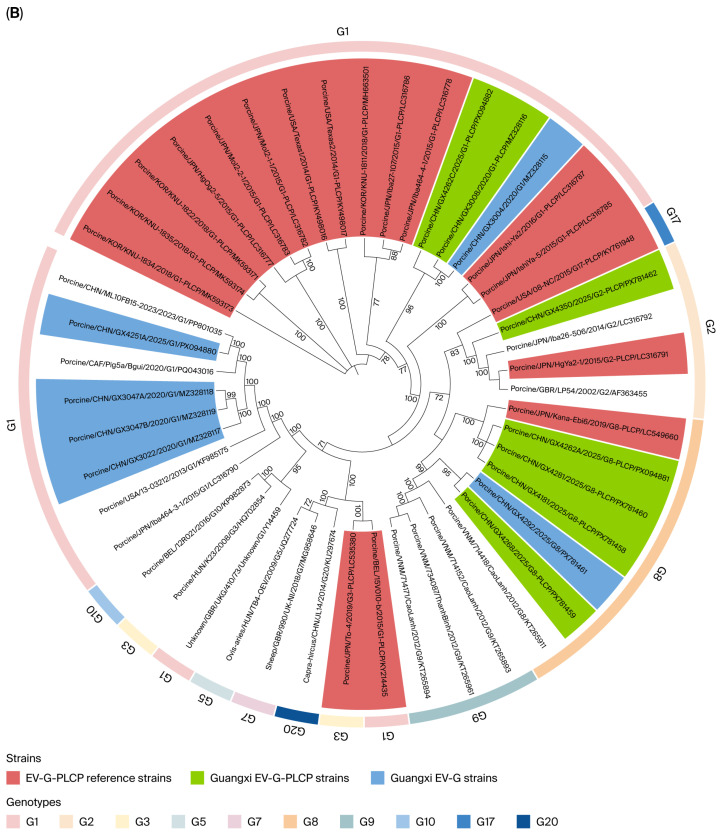
Phylogenetic analysis of Guangxi EV-G strains based on VP1 and complete-genome sequences. (**A**) Phylogenetic tree constructed based on VP1 gene sequences. (**B**) Phylogenetic tree constructed based on complete-genome sequences. Both trees were constructed using the ML method in MEGA 12 under the GTR+G+I model. Branch support was evaluated using 1000 bootstrap replicates, and bootstrap values greater than 70% are shown. The outer colored ring indicates genotype assignment. EV-G-PLCP reference strains are indicated in red, Guangxi EV-G-PLCP strains identified in this study are indicated in green, and Guangxi EV-G strains without PLCP insertions are indicated in blue.

A phylogenetic tree based on the VP1 gene sequences of 13 Guangxi EV-G strains together with 66 reference strains representing 20 genotypes (EV-G1–EV-G20) was constructed using the ML method in MEGA 12 (Figure 2A). The results showed that the 79 EV-G VP1 sequences could be divided into multiple phylogenetic branches.

Among the Guangxi strains, seven strains (GX3004, GX3008, GX3022, GX3047A, GX3047B, GX4251A, and GX4262C) clustered within the EV-G1 branch, one strain (GX4350) clustered within the EV-G2 branch, and the remaining five strains (GX4181, GX4262A, GX4268, GX4281, and GX4292) clustered within the EV-G8 branch, indicating that the EV-G strains identified in Guangxi in this study were mainly distributed among three genotypes, namely G1, G2, and G8.

Notably, the distribution of reference strains carrying PLCP insertion sequences was not completely consistent in the VP1-based phylogenetic tree. Some PLCP reference strains still clustered within their corresponding genotype branches; for example, certain G1-PLCP, G2-PLCP, and G17-PLCP reference strains were located within the G1, G2, and G17 branches, respectively. In contrast, other PLCP reference strains, particularly some G1-PLCP, G3-PLCP, and G8-PLCP strains, formed relatively independent branches or branch groups, indicating substantial genetic divergence at the VP1 sequence level.

#### 3.5.2. Phylogenetic Analysis Based on the Complete Genome

A phylogenetic tree was constructed using the same ML method in MEGA 12 based on the complete genome sequences of 13 Guangxi EV-G strains and 34 EV-G reference strains (Figure 2B). The results showed that the clustering pattern of the Guangxi EV-G strains in the complete genome-based tree was generally consistent with that observed in the VP1-based tree, and the branch assignments of most strains remained relatively stable between the two analyses.

Specifically, three Guangxi strains (GX3004, GX3008, and GX4262C) clustered within the same branch as 13 G1 reference strains carrying PLCP insertion sequences, among which GX3008 and GX4262C also harbored PLCP insertions. Another four Guangxi strains (GX3022, GX3047A, GX3047B, and GX4251A) clustered within a different branch together with 4 G1 reference strains lacking PLCP insertion sequences. GX4350 stably clustered within the EV-G2 branch, whereas the remaining five strains (GX4181, GX4262A, GX4268, GX4281, and GX4292) clustered within the EV-G8 branch.

In addition, the reference strains KY214435 and LC535380 formed a relatively independent branch in the complete genome-based phylogenetic tree. Taken together, the VP1-based and complete genome-based phylogenetic analyses showed that, although their overall topological structures were largely consistent, certain strains, particularly some reference strains carrying PLCP insertion sequences, differed in their clustering positions between the two trees. This suggests that some strains may harbor recombination signals, which were further confirmed by the subsequent recombination analysis.

### 3.6. Molecular Characteristics of PLCP Insertion Sequences

Based on complete-genome alignment and comparison of the 2C/3A junction region, PLCP gene insertions were identified in seven of the 13 Guangxi EV-G strains (Table 3): strain GX3008 contained a 642-nt PLCP insertion at nt 5053–5694; GX4181 contained a 573-nt insertion at nt 5053–5625; GX4262A and GX4262C both contained a 573-nt insertion at nt 5054–5626; GX4268 and GX4281 both contained a 588-nt insertion at nt 5054–5641; and GX4350 contained a 573-nt insertion at nt 5057–5629.

Homology analysis of the PLCP gene sequences inserted in these seven Guangxi EV-G strains showed that the nucleotide identities ranged from 78.36% to 89.63%. Among them, GX3008 and GX4350 showed the lowest nucleotide identity (78.36%), whereas GX4268 and GX4281 showed the highest nucleotide identity (89.63%). The corresponding deduced amino acid identities ranged from 72.43% to 94.39%, with the lowest identity observed between GX3008 and GX4262C (72.43%) and the highest between GX4268 and GX4281 (94.39%).

Further comparative analysis between the seven Guangxi strains and 18 EV-G-PLCP reference strains showed that, among all 25 strains, the nucleotide identities of the PLCP insertion sequences ranged from 68.81% to 99.55%. The lowest nucleotide identity was observed between GX4350 and strain 15V010 b (68.81%), whereas the highest was observed between Texas2 and Texas1 (99.55%). The corresponding amino acid identities ranged from 66.82% to 99.55%, with the lowest identity observed between GX4262C and strain 15V010 b (66.82%) and the highest between HgOg2-5 and HgYa2-1 (99.55%).

In addition, the PLCP gene sequences of Guangxi EV-G strains GX3008, GX4181, GX4262A, GX4262C, GX4268, GX4281, and GX4350 were compared with those of PToV reference strains. The nucleotide identities ranged from 59.43% to 64.50%, and the deduced amino acid identities ranged from 52.78% to 56.13%. Compared with BToV reference strains, the nucleotide identities ranged from 58.65% to 63.41%, and the amino acid identities ranged from 51.39% to 55.19%. Overall, these results indicate that the PLCP insertion sequences carried by the Guangxi EV-G strains share relatively high homology with those of previously reported EV-G-PLCP strains, but only moderate homology with PLCP sequences derived from toroviruses.

**Figure 3:**
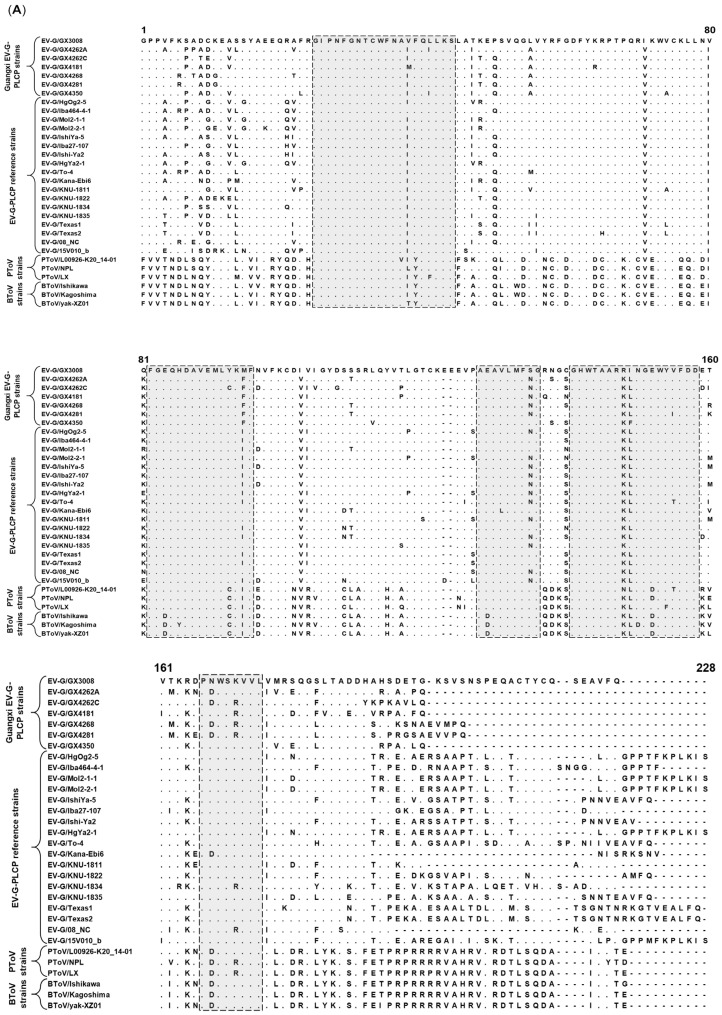

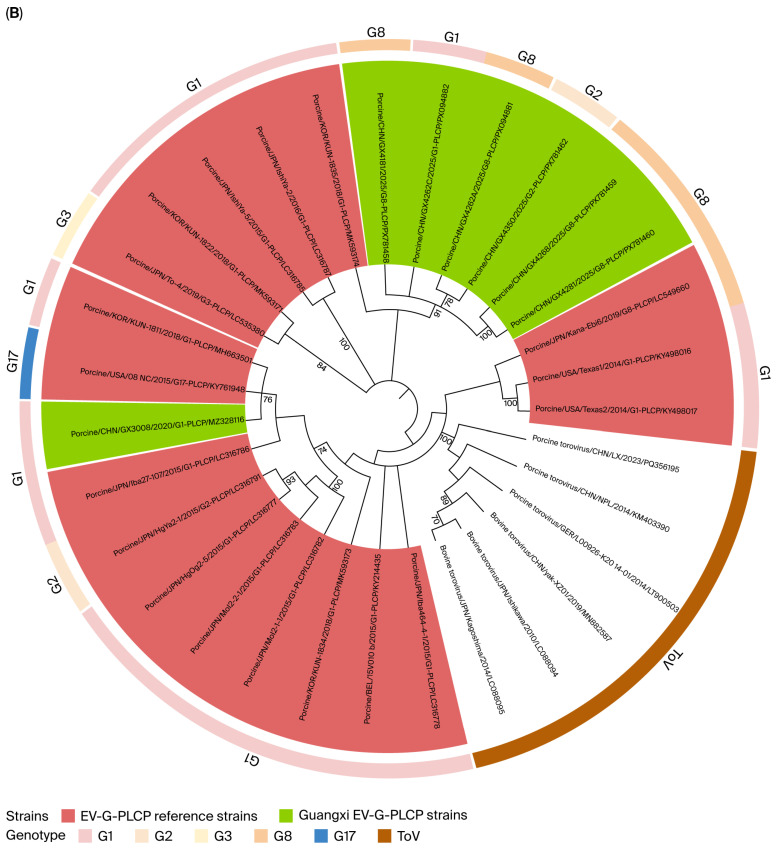
Amino acid sequence alignment and phylogenetic analysis of PLCP insertion sequences in Guangxi EV-G strains. (**A**) Multiple alignment of the PLCP amino acid sequences of seven Guangxi EV-G-PLCP strains, 18 EV-G-PLCP reference strains, and porcine torovirus (PToV) and bovine torovirus (BToV) reference strains, based on MAFFT alignment output and generated in MEGA 12. For clarity, the alignment is shown in three sections, corresponding to amino acid positions 1–80, 81–160, and 161–228 of the aligned PLCP insertion region. The complete amino acid sequence of GX3008 is shown as the reference sequence. In the remaining sequences, dots (.) indicate amino acid residues identical to those of GX3008, whereas letters indicate amino acid differences relative to GX3008. Vertical brackets indicate Guangxi EV-G-PLCP strains, EV-G-PLCP reference strains, PToV strains, and BToV strains. Gray shaded regions indicate putative conserved PLCP-related regions inferred from amino acid sequence alignment and comparison with previously reported EV-G-PLCP and torovirus PLCP reference sequences. (**B**) Phylogenetic tree constructed based on deduced PLCP amino acid sequences using the ML method in MEGA 12 under the JTT+G model. Branch support was evaluated using 1000 bootstrap replicates, and bootstrap values greater than 70% are shown. In Figure 3B, EV-G-PLCP reference strains are indicated in red, Guangxi EV-G-PLCP strains identified in this study are indicated in green, and the outer colored ring indicates genotype or torovirus group assignment.

Multiple alignment of PLCP amino acid sequences (Figure 3A) showed that the PLCP insertion sequences carried by the seven Guangxi EV-G strains shared a considerable number of conserved amino acid residues and putative conserved PLCP-related regions with previously reported EV-G-PLCP reference strains, whereas they differed markedly from those of PToV and BToV reference strains.

Phylogenetic analysis based on PLCP amino acid sequences (Figure 3B) showed that the PLCP sequences of all seven Guangxi EV-G strains clustered within the EV-G-PLCP clade and were clearly separated from the clades containing porcine torovirus and bovine torovirus PLCP sequences. Specifically, GX4268 and GX4281 clustered together, GX4262A showed a relatively close relationship with GX4350, GX3008 clustered with some G1-PLCP reference strains, and GX4262C and GX4181 were located at different positions within the same branch. These findings suggest that the PLCP insertion sequences carried by Guangxi EV-G strains are closely related to those of previously reported EV-G-PLCP strains, while also exhibiting a certain degree of genetic divergence among different strains.

### 3.7. Potential Recombination Events in Guangxi EV-G Strains

Recombination analysis was performed using RDP4.101 on the complete genome sequences of the 13 Guangxi EV-G strains together with 34 EV-G reference complete genome sequences retrieved from the GenBank database. After combined evaluation using RDP4 and SimPlot analyses, three potential recombination events with clearer support were retained, involving Guangxi strains GX3008, GX3022, and GX4292 (Table 4). All three retained events were supported by seven methods implemented in RDP4, namely RDP, Chimaera, BootScan, 3Seq, GENECONV, MaxChi, and SiScan.

**Figure 4:**
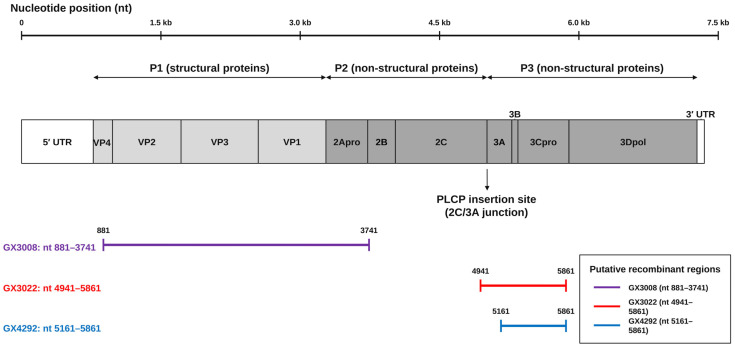
Schematic representation of the EV-G genome organization and the inferred recombinant regions identified in GX3008, GX3022, and GX4292. The schematic genome organization was illustrated with reference to the EV-G1 prototype strain UKG/410/73 (GenBank accession no. Y14459.1) and is intended to show the approximate locations of the viral protein-coding regions. The purple, red, and blue horizontal bars indicate the inferred recombinant regions of GX3008 (nt 881–3741), GX3022 (nt 4941–5861), and GX4292 (nt 5161–5861), respectively. The nucleotide coordinates of the recombinant regions correspond to the respective Guangxi EV-G strains. The PLCP insertion site is indicated at the 2C/3A junction.

**Figure 5:**
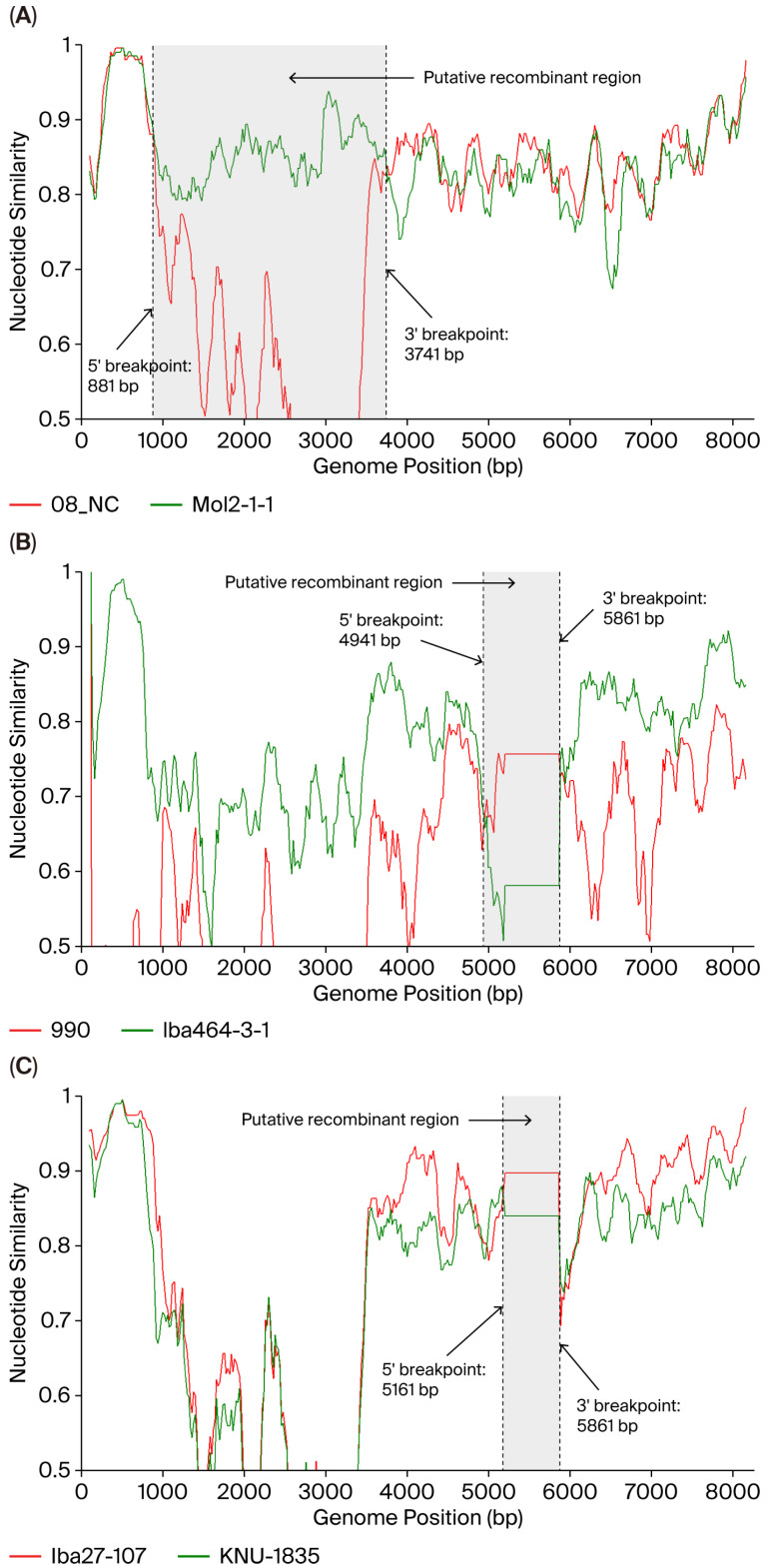
SimPlot analysis of three retained potential recombination events identified in Guangxi EV-G strains. (**A**) SimPlot analysis of the potential recombination event in GX3008, with 08 NC as the major parental strain and Mol2-1-1 as the minor parental strain. (**B**) SimPlot analysis of the potential recombination event in GX3022, with 990/UK-NI as the major parental strain and Iba464-3-1 as the minor parental strain. (**C**) SimPlot analysis of the potential recombination event in GX4292, with Iba27-107 as the major parental strain and KNU-1835 as the minor parental strain. The x-axis indicates genome position, and the y-axis indicates nucleotide similarity. Red and green curves indicate the putative major and minor parental strains, respectively. The gray shaded region indicates the putative recombinant region, which is delimited by the 5′ and 3′ breakpoints. The 5′ breakpoint, 3′ breakpoint, and putative recombinant region were determined with reference to the RDP4 output and interpreted together with the parental-similarity shifts observed in the corresponding SimPlot plots. SimPlot analysis was performed using a sliding window size of 200 bp and a step size of 20 bp.

The locations of the three inferred recombinant regions were illustrated in the context of the EV-G genome organization (Figure 4). These three potential recombination events were further evaluated using SimPlot v3.5.1 (Figure 5A–C). For GX3008, 08 NC was identified as the major parental strain, and Mol2-1-1 was identified as the minor parental strain. For GX3022, the major parental strain was 990/UK-NI, and the minor parental strain was Iba464-3-1. For GX4292, the major parental strain was Iba27-107, and the minor parental strain was KNU-1835. The 5′ breakpoint, 3′ breakpoint, and putative recombinant region of each event were determined with reference to the RDP4 output and interpreted together with the parental-similarity shifts observed in the corresponding SimPlot plots (Table 4).

Taken together, these results suggest that multiple potential recombination events occurred among Guangxi EV-G strains.

### 3.8. Selection Pressure Characteristics of the EV-G VP1 Gene

Selection pressure analysis was performed on 79 complete VP1 coding-region sequences, including 13 Guangxi EV-G strains and 66 reference strains representing 20 genotypes (EV-G1–EV-G20), using the SLAC, FEL, FUBAR, and MEME methods implemented in HYPHY v2.5.70. The results showed that only the MEME method identified seven potential positively selected sites, whereas no positive selection signals were detected by the SLAC, FEL, or FUBAR methods. Taken together, these findings indicate that the EV-G VP1 gene did not exhibit clear evidence of sustained positive selection.

Further analysis of the 79 complete VP1 coding-region sequences using DnaSP v6.12.03 showed that the nonsynonymous substitution rate (dN) was 0.20618, the synonymous substitution rate (dS) was 0.74285, and the ratio ω (dN/dS) was 0.27755. These results indicate that the VP1 gene of EV-G is overall under negative (purifying) selection, suggesting a relatively high degree of sequence conservation.

## 4. Discussion

### 4.1. Genetic Diversity and Genotype Distribution of EV-G

Since the isolation of the prototype strain PEV-9 in the United Kingdom in 1973, EV-G has spread widely in swine populations worldwide and has become one of the important enteric viruses persistently circulating in intensive pig production systems [13,28]. In the present study, systematic surveillance of clinical samples collected in Guangxi, a major pig-producing region in southern China, from 2020 to 2025 showed an overall EV-G positivity rate of 20.51%, which is comparable to the levels reported in Thailand, Japan, and other East Asian countries and regions, suggesting that EV-G has become relatively well established in this region [5,8,9].

Regarding genotype distribution, the 13 Guangxi EV-G strains identified in this study were mainly classified into three genotypes, G1, G2, and G8, with G1 being the predominant genotype. This finding is generally consistent with previous reports from Vietnam and some regions of China indicating that G1 is a major circulating genotype [3,6,29], and also suggests that EV-G strains circulating in Guangxi display a certain degree of genotype diversity [5,8,9,13]. Notably, genotypes G2 and G8 were also detected in this study, indicating that EV-G circulation in Guangxi is not restricted to a single genotype but instead exhibits a molecular epidemiological pattern characterized by the co-circulation of multiple genotypes [5,6,8,9].

Phylogenetic analysis further showed that, in the VP1-based tree, some reference strains carrying PLCP insertion sequences did not cluster strictly according to the conventional genotype pattern, but instead formed relatively independent branches or branch groups, indicating genetic divergence at the VP1 sequence level [5,9]. By contrast, although the complete genome-based phylogenetic tree was generally consistent with the VP1-based tree, some PLCP-carrying reference strains still differed in their clustering positions between the two trees, suggesting possible recombination signals [5,6]. Overall, the co-circulation of genotypes G1, G2, and G8 in Guangxi further highlights the molecular diversity of EV-G in this region and provides an important basis for further investigation of intra-species recombination signals and cross-genotype genetic variation.

### 4.2. Cross-Genotype Distribution of PLCP Insertions and Their Molecular Significance

In the present study, PLCP gene insertions of 573–642 nt were identified in Guangxi EV-G strains belonging to three distinct genotypes, G1, G2, and G8. Although PLCP insertions have previously been reported in several EV-G genotypes in different countries and regions [5,7,9,11,16,17], their simultaneous detection across multiple genotype backgrounds within a single geographic region has been less extensively documented. The present findings therefore provide direct regional evidence that PLCP insertions are not restricted to a single EV-G genotype and can be maintained in genetically distinct EV-G lineages co-circulating within the same region. This cross-genotype distribution further highlights the genomic plasticity of EV-G and suggests that PLCP acquisition or maintenance may occur under diverse viral genetic backgrounds.

In terms of sequence characteristics, the seven Guangxi EV-G strains carrying PLCP insertions exhibited a certain degree of heterogeneity, as reflected by the broad ranges of nucleotide and amino acid identities of their PLCP insertion sequences. Compared with previously reported EV-G-PLCP reference strains, the PLCP sequences carried by these Guangxi strains showed overall high homology, while still displaying a certain degree of divergence [5,18]. Phylogenetic analysis showed that the PLCP sequences of all seven Guangxi strains clustered within the EV-G-PLCP clade and were clearly separated from the clades containing porcine torovirus and bovine torovirus PLCP sequences [17,18]. Specifically, GX4268 and GX4281 clustered together, GX4262A was closely related to GX4350, whereas GX3008, GX4262C, and GX4181 occupied different positions within the same branch, suggesting that the PLCP insertion sequences carried by different Guangxi strains have undergone a certain degree of divergence.

Taken together, the occurrence of PLCP insertions in G1, G2, and G8 strains within Guangxi, together with the observed differences in insertion length, sequence homology, and phylogenetic position, suggests that these insertions are unlikely to be explained solely by the local transmission of a single PLCP-carrying ancestral strain. Instead, they may reflect the acquisition or maintenance of PLCP insertions under different genotype backgrounds followed by subsequent sequence divergence [5,11]. The cross-genotype distribution observed within a single geographic region provides further evidence of the genomic plasticity of EV-G. However, because this study was based on sequence and phylogenetic analyses, the biological function and adaptive significance of PLCP insertions remain to be experimentally validated.

### 4.3. Recombination Characteristics and Genomic Plasticity

Recombination is an important contributor to the genetic diversity of RNA viruses and EV-G [30,31]. Based on combined RDP4 and SimPlot analyses, three potential recombination events with clearer support were retained among the 13 Guangxi EV-G strains, involving GX3008, GX3022, and GX4292. All three retained events were supported by seven methods implemented in RDP4 and showed parental-similarity shifts in the corresponding SimPlot plots, suggesting the presence of potential recombination signals among Guangxi EV-G strains. Because the retained events were limited in number and involved different genomic intervals, no clearly conserved recombination hotspot could be defined based on the current dataset. The inferred recombinant region of GX3008 spanned the structural protein-coding region and extended into the non-structural protein-coding region, whereas the recombinant regions identified in GX3022 and GX4292 were mainly located within the non-structural protein-coding region. Recombination involving structural protein-coding regions may potentially affect capsid-related characteristics, whereas recombination in non-structural protein-coding regions may influence viral replication, polyprotein processing, or virus–host interactions. However, these potential biological consequences cannot be determined from sequence-based recombination analysis alone and require further functional validation.

Comparison of the VP1-based and complete genome-based phylogenetic trees further showed that some strains, particularly certain reference strains carrying PLCP insertion sequences, differed in their clustering positions between the two trees, which is consistent with the presence of potential recombination signals. Because different genomic regions may be subject to different functional constraints, the phylogenetic signals reflected by different genomic regions may not be entirely consistent, which represents an important molecular hallmark of recombination events [32,33]. Taken together with the three retained potential recombination events identified in this study, these findings suggest that recombination may have contributed to the genomic diversity of EV-G strains circulating in Guangxi [30,33].

Notably, PLCP-positive strains not only exhibited differences in insertion length, but also showed genetic heterogeneity in complete genome-based phylogenetic analysis and potential recombination-related signals. This observation is consistent with recent reports showing that EV-G can harbor diverse PLCP-related recombinant genome forms, including type 1 and type 2 recombinant EV-Gs [34]. This suggests that PLCP insertions may be associated with the genomic plasticity of EV-G [14]. These findings suggest that local insertion and recombination events may contribute to EV-G genomic diversity and plasticity [14,33].

### 4.4. Selection Pressure and Molecular Evolutionary Characteristics

The results of the selection pressure analysis provide further evidence for understanding the molecular evolutionary characteristics of EV-G. Analysis of 79 complete VP1 coding-region sequences in this study showed that only the MEME method detected seven potential positively selected sites, whereas no obvious positive selection signals were identified by the SLAC, FEL, or FUBAR methods. In addition, the overall dN/dS ratio of the VP1 gene was 0.27755, indicating that it is generally under negative (purifying) selection [13]. These findings suggest that, as the major structural protein-coding region, VP1 is relatively conserved and may be constrained by the need to maintain capsid structural stability.

Compared with VP1, the PLCP insertion sequences exhibited greater variability among different strains. Considering their sequence divergence, insertion-length variation, and phylogenetic differentiation, these findings suggest that the PLCP region may show sequence divergence after integration into the EV-G genome. Since PLCP-like sequences are related to torovirus PLCP genes with reported deubiquitinating and deISGylating activities, their potential biological significance in EV-G may deserve further investigation [14,15]. Although site-specific selection pressure analysis was not directly conducted for the PLCP-coding region in this study, the current results suggest that the structural VP1 region and the exogenous PLCP insertion region may be subject to different molecular constraints: VP1 appears to be generally conserved under purifying selection, whereas the PLCP insertion region shows greater sequence variability [30,35].

Taken together, the current results suggest that different genomic regions of EV-G may show distinct molecular characteristics. The structural protein-coding region appears to be relatively conserved, whereas the non-structural region, particularly the inserted fragment, may contribute to genomic plasticity [33,36]. This molecular pattern may help explain how EV-G maintains its basic genomic framework while acquiring additional sequence variation [16,37].

### 4.5. Study Limitations

This study still has several limitations. First, the samples analyzed in this study were mainly derived from routine clinical submissions from pigs suspected of EV-G infection. Therefore, the dataset was not generated through a standardized population-based sampling strategy, and detailed metadata such as pig age, farm structure, and herd-level information were not consistently available. Other porcine enteric viruses or mixed infections were not systematically screened; therefore, no conclusions regarding EV-G co-infection patterns can be drawn from the current dataset. In addition, the complete-genome dataset was limited to samples that met the sequencing-quality and genome-completeness criteria, which may have introduced sequencing-quality-related selection bias. Future studies with broader and more systematic sampling designs are needed to more accurately evaluate the epidemiological distribution of EV-G in Guangxi and other pig-producing regions. Second, this study was mainly based on phylogenetic, homology, recombination, and selection pressure analyses, and direct functional validation of the biological significance of PLCP insertions is still lacking. In addition, independent selection pressure analysis was not performed for the PLCP insertion region because of the limited number of available PLCP insertion sequences and their length variation. Future studies combining broader-scale surveillance and functional investigations are still needed to further clarify the biological significance of PLCP insertions in EV-G infection and evolution.

## 5. Conclusions

In this study, the circulation of EV-G in Guangxi, southern China, from 2020 to 2025 was systematically investigated, and complete genome sequences of 13 Guangxi EV-G strains were obtained. The results showed that the EV-G strains circulating in Guangxi were mainly distributed among three genotypes, G1, G2, and G8, with G1 being the predominant genotype. In addition, PLCP gene insertions located in the 2C/3A junction region were detected in seven strains, with insertion lengths ranging from 573 to 642 nt, involving genotypes G1, G2, and G8. Phylogenetic analysis showed that the PLCP sequences carried by the Guangxi strains clustered within the EV-G-PLCP clade and were clearly separated from the torovirus PLCP clade. Recombination analysis retained three potential recombination events with clearer combined support from RDP4 and SimPlot analyses, involving Guangxi strains GX3008, GX3022, and GX4292, while selection pressure analysis indicated that the VP1 gene is overall under negative selection.

Overall, this study demonstrates that EV-G circulating in Guangxi is characterized by the co-circulation of multiple genotypes, the cross-genotype distribution of PLCP insertions, and several potential recombination events. These findings provide new evidence for understanding the genetic diversity, genomic plasticity, and regional molecular characteristics of EV-G, and also provide an important basis for future functional validation of PLCP-related features and continued surveillance of EV-G in Guangxi.

## Figures and Tables

**Table 1:** Primer sequences used for reverse transcription-polymerase chain reaction (RT-PCR) detection of Enterovirus G (EV-G).

	Primer Name	Primer Sequence (5′–3′)	Template Position (nt)	Amplicon Size (bp)
Detection primers	EV-G-F	CAAGCACTTCTGTCTCCCCGG	200—220	314
	EV-G-R	GTTAGGATTAGCCGCATTCA	494—513	

**Table 2:** RT-PCR detection of EV-G in clinical samples from Guangxi, 2020–2025.

Region	No. of Samples Tested	No. of Positive Samples	Positivity Rate (%)
Beihai	13	2	15.38
Chongzuo	38	6	15.79
Fangchenggang	3	2	66.67
Guigang	20	1	5
Guilin	20	0	0
Hechi	10	0	0
Laibin	3	3	100
Liuzhou	22	7	31.82
Nanning	189	45	23.81
Qinzhou	4	1	25
Yulin	34	6	17.65
Total	356	73	20.51

**Table 3:** Sampling information, genotypes, genome lengths, PLCP insertions, and GenBank accession numbers of the 13 Guangxi EV-G strains.

Strain	Sampling Region	Sample Type	Collection Year	Genotype	Genome Length (nt)	PLCP Insertion (nt)	GenBank Accession
GX3004	Nanning	Intestinal contents	2020	G1	7391	—	MZ328115
GX3008	Nanning	Feces	2020	G1	8033	642	MZ328116
GX3022	Nanning	Intestinal contents	2020	G1	7354	—	MZ328117
GX3047A	Nanning	Intestinal tissue	2020	G1	7354	—	MZ328118
GX3047B	Nanning	Intestinal tissue	2020	G1	7354	—	MZ328119
GX4251A	Nanning	Feces	2025	G1	7356	—	PX094880
GX4262A	Fangchenggang	Feces	2025	G8	7965	573	PX094881
GX4262C	Fangchenggang	Feces	2025	G1	7965	573	PX094882
GX4181	Guigang	Feces	2025	G8	7964	573	PX781458
GX4268	Liuzhou	Feces	2025	G8	7980	588	PX781459
GX4281	Nanning	Feces	2025	G8	7980	588	PX781460
GX4292	Nanning	Feces	2025	G8	7391	—	PX781461
GX4350	Nanning	Feces	2025	G2	7971	573	PX781462

Note: —, no PLCP insertion detected.

**Table 4:** Potential recombination events retained after combined RDP4 and SimPlot evaluation in Guangxi EV-G strains.

No.	Recombinant EV-G Strain	Major Parental Strain	Minor Parental Strain	5′ Breakpoint	3′ Breakpoint	Putative Recombinant Region
1	Porcine/CHN/GX3008/2020/G1-PLCP/MZ328116	Porcine/USA/08_NC/2015/G17-PLCP/KY761948	Porcine/JPN/Mol2-1-1/2015/G1-PLCP/LC316782	881	3741	881–3741
2	Porcine/CHN/GX3022/2020/G1/MZ328117	Sheep/GBR/990/UK-NI/2018/G7/MG958646	Porcine/JPN/Iba464-3-1/2015/G1/LC316790	4941	5861	4941–5861
3	Porcine/CHN/GX4292/2025/G8/PX781461	Porcine/JPN/Iba27-107/2015/G1-PLCP/LC316786	Porcine/KOR/KNU-1835/2018/G1-PLCP/MK593174	5161	5861	5161–5861

## Data Availability

The data presented in this study are openly available in GenBank [https://www.ncbi.nlm.nih.gov/genbank (accessed on 20 March 2026)] [MZ328115, MZ328116, MZ328117, MZ328118, MZ328119, PX094880, PX094881, PX094882, PX781458, PX781459, PX781460, PX781461, PX781462].
